# Mechanisms of host manipulation by *Neisseria gonorrhoeae*

**DOI:** 10.3389/fmicb.2023.1119834

**Published:** 2023-02-03

**Authors:** Emma Walker, Stacy van Niekerk, Kyrin Hanning, William Kelton, Joanna Hicks

**Affiliations:** ^1^Te Huataki Waiora, School of Health, University of Waikato, Hamilton, New Zealand; ^2^Te Aka Mātuatua School of Science, University of Waikato, Hamilton, New Zealand

**Keywords:** gonorrhoea (*Neisseria gonorrhoeae*), apoptosis, cell signaling and infection, gonorrhoea pathogenesis, immunology and infectious disease

## Abstract

*Neisseria gonorrhoeae* (also known as gonococcus) has been causing gonorrhoea in humans since ancient Egyptian times. Today, global gonorrhoea infections are rising at an alarming rate, in concert with an increasing number of antimicrobial-resistant strains. The gonococcus has concurrently evolved several intricate mechanisms that promote pathogenesis by evading both host immunity and defeating common therapeutic interventions. Central to these adaptations is the ability of the gonococcus to manipulate various host microenvironments upon infection. For example, the gonococcus can survive within neutrophils through direct regulation of both the oxidative burst response and maturation of the phagosome; a concerning trait given the important role neutrophils have in defending against invading pathogens. Hence, a detailed understanding of how *N. gonorrhoeae* exploits the human host to establish and maintain infection is crucial for combating this pathogen. This review summarizes the mechanisms behind host manipulation, with a central focus on the exploitation of host epithelial cell signaling to promote colonization and invasion of the epithelial lining, the modulation of the host immune response to evade both innate and adaptive defenses, and the manipulation of host cell death pathways to both assist colonization and combat antimicrobial activities of innate immune cells. Collectively, these pathways act in concert to enable *N. gonorrhoeae* to colonize and invade a wide array of host tissues, both establishing and disseminating gonococcal infection.

## 1. Introduction

Gonorrhoea is one of the most ancient human diseases, with written accounts dating back as far as 1550 BC (Nickel, 2005). This sexually transmitted infection has since propagated into a global public health crisis today. Strains of the causative agent, *Neisseria gonorrhoeae* (also known as gonococcus), have acquired resistance to every antibiotic used to effectively treat the disease since the late 1930s (Unemo and Shafer, 2011); and in 2018, the first treatment failure of front-line antibiotics was reported in the United Kingdom (Eyre et al., 2018). Alongside this threat of antibiotic resistance, is the continued rise in global case numbers (Kirkcaldy et al., 2019; Unemo et al., 2019; Whelan et al., 2021) and the lack of an effective vaccine against gonococcal infection (McIntosh, 2020). Together, this highlights the pressing need for gonococcal-focused research to prevent the emergence of untreatable gonorrhoea in the near future.

*N. gonorrhoeae* is an obligate human pathogen, and predominantly infects the mucosal epithelium of the human urogenital tract, but is also capable of infecting other exposed mucosal sites, such as the rectum, pharynx, and conjunctiva (Quillin and Seifert, 2018). Gonococcal infection can present in both women and men as symptomatic or asymptomatic, with higher rates of asymptomatic infection reported in women (Unemo et al., 2019); although, asymptomatic infection rates in men have been estimated to be as high as 40% (Martín-Sánchez et al., 2020). The significant proportion of asymptomatic infections caused by the gonococcus, generally leaves the individual unaware of the infection they harbor, increasing the chances of both transmission and dissemination. Symptomatic infection usually presents as urethritis in males and as ectocervicitis or endocervicitis in females. Although disseminated infection to other anatomic locations occurs only in a minority of cases, extensive localized infection can cause severe complications, including pelvic inflammatory disease, infertility, ectopic pregnancy, or neonatal blindness resulting from transmission during birth. Moreover, gonococcal infection also increases the chances of the individual contracting additional sexually transmitted infections, such as HIV (Masi and Eisenstein, 1981; Sandström, 1987; Little, 2006; Quillin and Seifert, 2018). There is little information regarding the outcome of asymptomatic infections as individuals do not seek treatment. However, anecdotal evidence suggests asymptomatic infection generally self-resolves within several months, although, in extreme cases the infection may persist for many years (Russell and Hook, 2009). This parallels the observation that symptomatic infections also self-resolve in cases of treatment failures or in the absence of treatment, although the infection is thought to persist for at least 14 days (Liu et al., 2014; Lovett and Duncan, 2019).

Given the wide array of host mucosa that *N. gonorrhoeae* can infect, it is not surprising that the bacterium possesses an array of surface molecules to allow entry and invasion of these various types of tissue (Quillin and Seifert, 2018). In addition, the gonococcus possesses an extraordinary ability to undergo both phase and antigenic variation of these primary surface antigens with extremely high turn-over rates (Virji, 2009). In part, this feature of the gonococcus explains why this bacterium has remained such a prominent human pathogen as it is exceedingly difficult for the human immune system to develop immunological memory. *N. gonorrhoeae* expresses three primary outer membrane proteins, the porin ion channel protein (PorB), the colony opacity-associated (Opa) proteins, and the reduction modifiable protein (Rmp); in addition to two key pathogen-associated molecular patterns (PAMPs), lipooligosaccharides (LOS) and the type IV pili (Edwards and Apicella, 2004). The majority of these virulence factors have the ability to undergo phase and/or antigenic variation (Stern et al., 1986; Burch et al., 1997; Criss et al., 2005), with the exception of Rmp (Judd, 1982) and the abundant outer membrane protein, PorB, where all gonococcal strains express one of the PorB isotypes – PorB_IA_ or PorB_IB_ (Derrick et al., 1999). Despite the antigenic-stable nature of PorB within a given strain, allelic variation between gonococcal strains is common among the PorB isotypes, and occurs in the surface-exposed loops of the porin molecules (Fudyk et al., 1999; Garvin et al., 2008). In terms of antigenic variation, a single strain of *N. gonorrhoeae* can encode up to 11 distinct *opa* genes, allowing for the constitutive expression of several distinct Opa proteins (Stern and Meyer, 1987; Bhat et al., 1991). Furthermore, a pentameric pyrimidine motif (CTCTT) is located in the 5′ region of all gonococcal *opa* loci, with varying numbers of the repeating unit per locus. This motif is susceptible to slip-strand misrepair, causing either the deletion or insertion of this repeating unit, potentially leading to out-of-frame transcripts and subsequently no Opa expression (Stern et al., 1986). Antigenic variation of the type IV pili system stems from homologous recombination of the single *pilE* locus, with portions of the 19 promoterless (or “silent”) variations of this gene (the *pilS* loci), which lie upstream of *pilE* (Hamrick et al., 2001; Sechman et al., 2005). Variation of the LOS molecules occurs indirectly through phase variation of the cytosolic glycosyltransferases, which enzymatically determine the glycan profile of the LOS molecules (Danaher et al., 1995; Burch et al., 1997).

In addition to constant surface variation, an additional key trait underpinning the success of the gonococcus is its ability to manipulate host cell signaling events in favor of its pathogenesis. Again, an unsurprising feat given the longevity of the relationship this bacterium has had with its human host. Inevitably, the primary mechanisms by which the gonococcus exerts this manipulation is through its key outer membrane antigens. Obtaining both a detailed and holistic understanding of these intricately evolved host manipulation events will greatly advance our fight against this high priority pathogen.

Here, we summarize the literature surrounding the host epithelial signaling pathways the gonococcus takes advantage of to both colonize and penetrate the epithelial lining of the urogenital tract to establish infection. Following this, we highlight the primary mechanisms used by the gonococcus to subsequently alter the host immune and cell death responses in a manner which favors pathogenesis. Notably, there appears to be a general consensus throughout the research community that both up- and down-regulation of the host immune and cell death pathways can promote gonococcal infection, likely dependent on the anatomical site and time-period of the infection. While the underlying detail behind these mechanisms remains largely uncharacterized, the induction of anti-inflammatory immune states and the establishment of intracellular niches by preventing cell death are ultimately essential for facilitating transmission. In contrast, an upregulation of proinflammatory responses during infection may allow the gonococcus to overcome epithelial barriers through tissue damage, gaining access to nutrients and deeper tissue, thus enabling further dissemination of the pathogen.

## 2. Exploitation of host epithelial cell signaling pathways to establish infection

Gonococci establish infection at the mucosal epithelia of the human genital tract using three interrelated events: colonization of the epithelia, invasion of epithelial cells, and trafficking into the subepithelial tissue. The mechanisms for each of these events differ between males and females, and within females at different anatomic locations. However, all involve close interactions with host cells and alteration of host cell signaling pathways, generally leading to decreased epithelial cell exfoliation to promote colonization or invasion into the epithelial layer.

### 2.1. Gonococcal interactions with male urethral epithelial cells

Gonococcal infection in men most often occurs as acute urethritis resulting from the inflammatory response to infecting gonococci (Ramsey et al., 1995). Generally, the hallmark of symptomatic gonorrhoea in men is the presence of a purulent discharge, associated with the influx of neutrophils and the shedding of urethral epithelial cells. Human studies have identified an incubation period of up to 40 h extending from the moment of infection to the onset of clinical symptoms, where gonococci cannot be cultured from the male urethra (Schneider et al., 1995). This suggests that the gonococci enter the protective environment of epithelial cells early in disease, enabling both survival and replication (Edwards and Apicella, 2004). While pili mediate initial cell-to-cell contact events essential for colonization, and Opa proteins promote gonococcal adherence, the concurrent binding of both pili and Opa proteins further promotes colonization and infection. The exact mechanism and signaling pathways affected upon binding to male urethral epithelial cells have only partially been deciphered. Although pili expression is a known requirement for invasion, the receptor which the pili engages with during male infection has not been identified *in vivo*. Known pili receptors include the complement receptors, CD46 and CR3, and other I-domain-containing integrins, such as the α1β1 and α2β1 integrins. CR3 expression is absent within the male urogenital epithelium, leaving CD46 and other I-domain-containing integrins as likely candidates. Although binding of pili to the complement regulatory protein, CD46, initiated infection in various cell types *in vitro* (Källström et al., 1997; Gill et al., 2003; Gill and Atkinson, 2004), and CD46 expression has been demonstrated in female genital tissue (Edwards et al., 2002), to date no pilus-CD46 interaction has been shown *in vivo* in male exudates (Edwards and Apicella, 2005). In support of this, the first interactions of the gonococcal pili with male urethral epithelial cells *in vitro*, demonstrated an early preference for binding the I-domain-containing integrins, α1β1 and α2β1, over CD46 to initiate gonococcal adherence and colonization (Edwards and Apicella, 2005). Gonococcal invasion of male urethral epithelial cells is facilitated by interactions of the asialoglycoprotein receptor (ASGP-R) with LOS on the gonococcal cell membrane, resulting in the formation of an actin pedestal underneath the bacterium (Apicella et al., 1996; Harvey et al., 1997). Following pedestal formation, ASGP-R-mediated endocytosis of the bacterium ensues, in both an actin- (Giardina et al., 1998) and clathrin-dependent (Harvey et al., 1997) manner, subsequently mediating cytoskeletal rearrangements to further promote gonococcal invasion and transcytosis.

### 2.2. Gonococcal interactions with epithelial cells of the female reproductive tract

In contrast to the male urogenital tract, the female reproductive tract presents a variety of epithelial cell types, from the vagina and ectocervix of the lower reproductive tract, to the endocervix, uterus, and fallopian tubes of the upper reproductive tract. The gonococci manipulate different host pathways by binding to differentially expressed receptors on the surface of different cervical epithelial cells to promote either colonization or cell invasion. Cells in the female reproductive tract transition from non-polarized squamous epithelial cells in the ectocervix, to single-layer, polarized columnar cells in the endocervix, with the area of cellular transition known as the transition zone (Prendiville and Sankaranarayanan, 2017). The monolayer epithelium of the endocervix differs in that it is held together by apical junctions compared to adherent junctions in the squamous epithelium of the ectocervix, which act to prevent pathogen entry and maintain polarity of the endocervical layer. Clinical observations and microscopy studies by Harkness in the 1940s demonstrated that during natural infection, *N. gonorrhoeae* was incapable of invading the ectocervix, but colonization of the endocervix led to transmigration into the subepithelium or lymphatic vessels (Harkness, 1948). Transmigration into the subepithelium has also been shown to occur within the transition zone (Yu et al., 2019). Furthermore, recent research using a human cervical tissue explant model demonstrated that expression of gonococcal Opa proteins promotes colonization in the ectocervical region of the female genital tract by preventing epithelial cell exfoliation. Early studies using organ cultures of human fallopian tubes demonstrated piliated gonococci result in sloughing of ciliated epithelial cells compared to non-piliated gonococci, highlighting the importance of pili in infection (McGee et al., 1981). This damage was not observed for non-ciliated epithelial cells, with gonococci attaching to these cells, leading to cell invasion and trafficking to the subepithelial tissue (see (Lenz and Dillard, 2018) for a review on *N. gonorrhoeae* pathogenesis of the human fallopian tubes). Similar to that within the male urethral epithelium, binding of pili, Opa, and PorB also activates host cell signaling pathways to promote colonization and infection within the female reproductive tract.

#### 2.2.1. Binding of the type IV pilus activates host epithelial cell signaling pathways to promote colonization and infection of the female reproductive tract

Attachment of the gonococcus to female epithelial cells is initiated by the type IV pilus. The pilus binds to the complement receptors, CD46 and CR3, and I-domain containing integrin receptors. CR3 is an I-domain containing receptor, where pilus binding activates the CR3 I-domain, leading to membrane ruffling, subsequently mediating cell invasion (Edwards et al., 2001). Together with membrane ruffling, this I-domain activation of CR3 modulates additional downstream signaling events to further facilitate infection of endocervical cells which have high expression of the CR3 receptor (Jennings et al., 2011). Binding of the pilus to CD46 does not occur at the early stages of infection, but CD46 clustering and colocalization with the gonococcus is observed at later time points in infection (>6 h; Källström et al., 1997; Gill et al., 2003; Gill and Atkinson, 2004). Binding to CD46 triggers phosphorylation of tyrosines on the CD46 cytosolic tail (Lee et al., 2002), and the subsequent release of intracellular Ca^2+^ (Ayala et al., 2005). Calcium release promotes intracellular survival by redirecting the core lysosomal membrane protein, Lamp1, to the plasma membrane where it is cleaved by the immunoglobulin A1 (IgA1) protease secreted by adherent gonococci, reducing the number of lysosomes in infected cells (Källström et al., 1997; Lin et al., 1997; Hopper et al., 2000; Ayala et al., 2001). To further promote infection, binding of pili to epithelial cells results in remodeling of the cytoskeleton and modulation of phagocytosis by activation of the phosphoinositide-3 (PI-3) kinase/Akt signaling pathway (Lee et al., 2005). Activation of this cascade triggers the epithelial cell to produce the lipid secondary messenger, [PI(3,4,5)P3], which activates Akt and the PI-3 kinase to be translocated to the outer face of the infected cell membrane. The subsequent close proximity of these kinases to the attached microcolony has been postulated to enable the gonococcus access to the lipid secondary messenger, which is known to affect bacterial motility and behavior, thereby enhancing colonization and possibly host cell invasion (Lee et al., 2005). In addition, the force generated from pilus retraction on host cells activates expression of infection-regulated genes known to be expressed upon mechanical stress, through activation of mitogen-activated protein kinase (MAPK), which influences the ability of the cell to survive apoptosis and is discussed later in this review (Howie et al., 2005). Retraction of the pilus upon binding to epithelial cells also plays an important role in the activation of NF-κB, a global transcription factor that controls transcription, cytokine production, and cell survival. The microcolony is critical for NF-κB activation during gonococcal infection, presumably by generating an increased retraction force on the epithelial cell membrane leading to increased NF-κB activation (Dietrich et al., 2011). Through regulation of this global transcription factor, gonococcal microcolonies have the ability to regulate a range of host cell processes, including apoptosis and the innate immune response, influencing how infection proceeds.

#### 2.2.2. Opa proteins interact with host cells to promote gonococcal pathogenesis by manipulation of cell signaling pathways

Pilus retraction by the gonococcus triggers a tight association between Opa outer membrane proteins on the gonococcus and host cell receptors (Merz et al., 1996, 2000). The importance of Opa proteins in infection is highlighted by the findings that Opa expression increases gonococcal fitness in the female reproductive tract of mice (Cole et al., 2010), and the presence of Opa antibodies in the blood of infected women correlates with a decreased risk of gonococcal salpingitis (Plummer et al., 1994). Additionally, *N. gonorrhoeae* isolates from naturally infected men and women are Opa^+^, as are isolates obtained post-infection of men with Opa^−^ colonies (James and Swanson, 1978; Jerse et al., 1994; Isbey et al., 1997). The majority of Opa proteins bind the carcinoembryonic antigen cell adhesion molecules (CEACAMs; Gray-Owen et al., 1997a; Sadarangani et al., 2011; Virji et al., 1996), however, the Opa50 isoform is also able to bind heparin sulfate proteoglycan (HSPG) receptors (van Putten and Paul, 1995; Freissler et al., 2000). Although, expression of the Opa50 variant has no significant effect on infection with any cervical cell types in tissue explant models (Yu et al., 2019). This could be due to HSPG receptors not being exposed on the apical surface of some epithelial tissues, therefore Opa interactions with HSPG do not mediate gonococcal attachment to the apical membrane (Lin et al., 1997), given HSPG binding does allow effective gonococcal uptake into non-polarized epithelial cells (Chen et al., 1995; van Putten and Paul, 1995; Freissler et al., 2000). The interaction of Opa proteins with HSPG also activates phosphatidylcholine-specific phospholipase C (PLC) and the acid sphingomyelinase, resulting in the release of diacylglycerol and ceramide, initiating signaling pathways to promote gonococcal invasion of epithelial cells (Grassmé et al., 1997).

Four different CEACAM receptors (CEACAM1, CEACAM3, CEACAM5, and CEACAM6) on various cell types can independently associate with gonococcal Opa proteins (for a review of gonococcal Opa proteins see Sadarangani et al., 2011). CEACAM1, CEACAM5, and CEACAM6 are expressed on the apical membrane of various epithelial cells, whereas CEACAM3 is expressed exclusively by neutrophils. CEACAMs are differentially recognized by Opa variants, and as such the expression and distribution of CEACAM receptors can influence gonococcal interactions with host cells. For example, epithelial cells of the female reproductive tract differentially express CEACAMs, with high expression in ecto- and endo-cervical cells and low expression in cells found in the transition zone. To date, there has been no study investigating CEACAM expression within epithelial cells of the male urethra, but a mouse model expressing human CEACAMs shows expression of CEACAM5 on the male urethral cell surface (Chan and Stanners, 2004).

**Figure 1:**
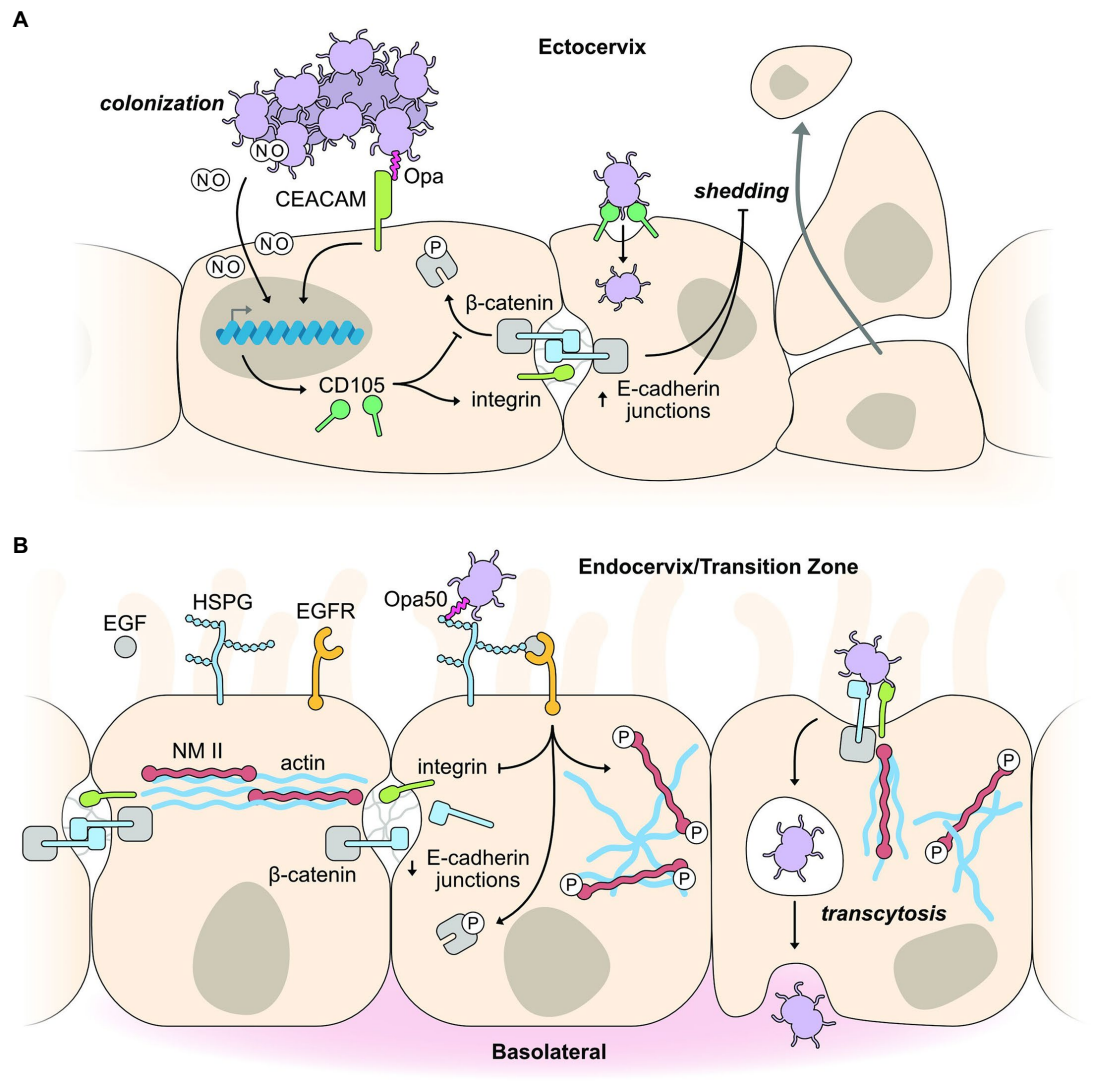
Exploitation of host epithelial cell signaling pathways for colonization and infection. **(A)** Binding of Opa-expressing gonococci to CEACAM receptors on squamous epithelial cells of the ectocervix promotes colonization by inhibiting epithelial cell shedding. Opa binding by CEACAM receptors triggers changes in host gene expression, including the expression of CD105, which activates integrin and decreases phosphorylation of the cell junction protein, β-catenin. Collectively, this strengthens and maintains the E-cadherin junction between epithelial cells leading to decreased epithelial cell shedding in response to gonococcal infection. Opa-CEACAM interactions result in a tight association between the gonococcus and host cell, allowing nitric oxide (NO) produced by the gonococci (under anaerobic respiration) to diffuse into the host cell, activating expression of genes, such as CD105, to decrease cell shedding as outlined above. **(B)** Binding of gonococci to endocervical cells of the transition zone initiates cell invasion by cytoskeletal rearrangements and weakening of cell junctions. CEACAM expression is reduced in endocervical cells of the transition zone. Therefore, gonococci interact with HSPG receptors leading to activation of the EGFR receptor. Activation of the associated signaling pathways results in decreased integrin expression, increased phosphorylation of β-catenin, and movement of E-cadherin junction proteins to the membrane under the gonococcus, acting in concert to weaken cell junctions. Phosphorylation of NMII results in its rearrangement alongside actin filaments to facilitate cell invasion.

Exfoliation of infected epithelial cells is often a first line defense mechanism of the innate immune system to prevent bacterial colonization. The interactions between CEACAM and Opa promote colonization of ecto- and endo-cervical cells by suppressing the exfoliation of mucosal cells (Figure 1). A high level of CEACAM expression in the lumen of the ectocervix drives strong colonization of Opa-expressing gonococci (Yu et al., 2019). Downstream CEACAM signaling increases integrin-β1 activity (Muenzner et al., 2010; Yu et al., 2019), which decreases epithelial cell shedding, promoting colonization, while also inhibiting β-catenin phosphorylation to stabilize cell-to-cell junctions (Figure 1) leading to reduced cell layer penetration (Yu et al., 2019). Binding of gonococci to CEACAM receptors also triggers the *de novo* expression of CD105, a member of the transforming growth factor-β1 receptor family, to promote integrin activity and enhance binding to extracellular matrix proteins leading to decreased exfoliation of epithelial cells, increasing gonococcal colonization (Muenzner et al., 2005, 2010; Figure 1).

It is well established that gonococcus binding to CEACAMs modulates cell signaling (Huber et al., 1999; Poy et al., 2002; Nagaishi et al., 2006; Leung et al., 2008). But how CEACAM clustering and binding at the cell membrane leads to altered gene expression was unknown until a recent study by Muenzner and Hauck (2020) identified an unexpected role for the soluble, diffusible gas nitric oxide (NO) in CD105 expression (Muenzner and Hauck, 2020). NO is produced by gonococci during anaerobic metabolism, and this membrane permeable gas initiates a conserved eukaryotic cell signaling pathway involving soluble guanylate cyclase (sGC), protein kinase G, and the transcription factor, CREB, to upregulate expression of CD105, causing increased cell-matrix adhesion and decreased epithelial cell exfoliation (Figure 1; Muenzner and Hauck, 2020). CEACAM clustering or CEACAM engagement by metabolically inactive bacteria does not lead to the downstream expression of CD105, highlighting the importance of metabolically active and anaerobic pre-conditioning of the gonococci to produce NO. The close interaction between gonococci and epithelial cells resulting from Opa-CEACAM interactions is a requirement to allow sufficient amounts of NO to reach the host cell cytoplasm, as although NO can transverse freely it can only act across short distances. The Opa-CEACAM interaction leads to a tight embedding of the bacteria into the host membrane or endocytic uptake into the host cell (Wang et al., 1998; Schmitter et al., 2007), allowing bacterial produced NO to reach the host cell cytoplasm in sufficient quantities to initiate NO-based signaling pathways. The production of NO in gonococci is dependent on the nitrate reductase protein, AniA, which is used in the switch to anaerobic respiration. AniA is one of the major proteins expressed under low oxygen conditions, such as those in the female genital tract. An interesting aside as to the importance of AniA indirectly leading to decreased epithelial cell exfoliation is the emergence of a novel group of *Neisseria meningitidis* that are capable of colonizing the urogenital tract and can transmit by sexual contact (Bazan et al., 2016). This ability to colonize the urogenital tract has been accompanied by gene loss, but also by the acquisition of a large gonococcal genomic fragment containing the AniA gene (Tzeng et al., 2017).

Low CEACAM expression in cells of the transition zone and endocervix abolish the effects of Opa binding, enabling gonococci to penetrate host cells regardless of which Opa variant they express. In a 2019 study utilizing female reproductive tissue explants, binding of Opa50 to HSPG receptors was found to induce phosphorylation of the cell junction associated protein, β-catenin, and reduce integrin expression to promote gonococci penetration of the epithelial layer (Figure 1; Yu et al., 2019). β-catenin is an adapter protein for the apical junction protein E-cadherin, and upon phosphorylation, induces the disassembly of this apical junction. Phosphorylated β-catenin is subsequently redistributed from the apical junction to the cytoplasm and gonococcal adherence sites to facilitate internalization (Edwards et al., 2013), while E-cadherin is also redistributed to the cytoplasm (Rodríguez-Tirado et al., 2012). These cell junction protein redistributions do not lead to a significant increase in the permeability of the epithelial layer, yet weakens the lateral mobility between the apical and basolateral membranes. The gonococcal-induced phosphorylation of β-catenin and its redistribution depends on the kinase activity of the epidermal growth factor receptor (EGFR; Edwards et al., 2013). EGFR activation appears not to depend on a specific surface molecule on the gonococcus but on a variety of different surface molecules which interact with different host receptors to induce the production of EGFR ligands for EGFR activation (Swanson et al., 2011). Although only a small percent of adherent gonococci invade and cross the epithelial cell layer, these gonococci can cause both local and disseminated infections. Gonococci cross the epithelial barrier primarily *via* an intracellular rather than an intercellular route, although they may gain access to deeper tissues *via* an intercellular route (McGee et al., 1981; Apicella et al., 1996; Mosleh et al., 1997).

Interactions of gonococcal surface molecules with the epithelial cell surface also activates host cell signaling cascades that trigger the reorganization of the actin cytoskeleton, which is required for host cell entry and transmigration across the epithelial layer. EGFR, as discussed above, is activated by gonococci binding and is a common surface receptor that is essential for epithelial cell survival and proliferation through the activation of several signaling cascades (PI3K, PLCγ, calcium flux, and PKC and MAP kinases), all of which can lead to actin rearrangement (Swanson et al., 2011; Figure 1). Gonococci induce disassembly of the actomyosin ring, which supports cell junction complexes by inducing calcium-dependent phosphorylation of nonmuscle myosin II (NMII), leading to activation and reorganization of NMII from cell junctions to gonococcal adherence sites (Wang et al., 2017). The reorganization of NMII to gonococcal adherence sites is part of the substantial rearrangements seen in the host cell directly beneath the points of bacterial attachment. Early studies demonstrated membrane perturbations resulting in membrane ruffles upon gonococci infection of primary ecto- and endo-cervical cells (Edwards et al., 2000). Actin-associated proteins, such as ezrin and vinculin, accumulate in response to gonococcal adhesion, further promoting the actin-dependent entry of primary epithelial cells (Edwards et al., 2000). Although, differences in the involvement of the actin cytoskeleton have been observed depending on which receptor is used for internalization.

#### 2.2.3. PorB interacts with host cell membranes, influencing host cell signaling to promote intracellular survival

PorB is the most abundant protein in the gonococcus and is essential for its viability (Britigan et al., 1985). The porin complex is a trimeric arrangement spanning the membrane, forming an anion selective channel. Gonococcal strains expressing the PorB_IA_ protein are more invasive (van Putten et al., 1998), and are often isolated from patients with disseminated gonococcal infection, while strains expressing PorB_IB_ tend to be associated with pelvic inflammatory disease, and are isolated from patients with localized gonococcal infection (Morello and Bohnhoff, 1989). Notably, the majority of gonococcal PorB research appears to be focused on the PorB_IB_ isoform; given the implications the PorB_IA_ isoform clearly has on disseminating infection, research efforts should be extended to include this more potent PorB protein. Like the pilus, PorB induces calcium transients in the host cell, leading to a reduction in the Lamp1 glycoprotein *via* gonococcal IgA1 protease cleavage, and a general reduction of lysosome numbers in infected cells (Lin et al., 1997; Hopper et al., 2000). These effects ultimately increase gonococcal intracellular survival and growth. The pilus and porin act in concert to induce calcium fluctuations in the host cell; an important secondary messenger that can influence cell growth, differentiation, motility, apoptosis, and necrosis (Ayala et al., 2005).

## 3. Modulation of the host immune response

**Figure 2:**
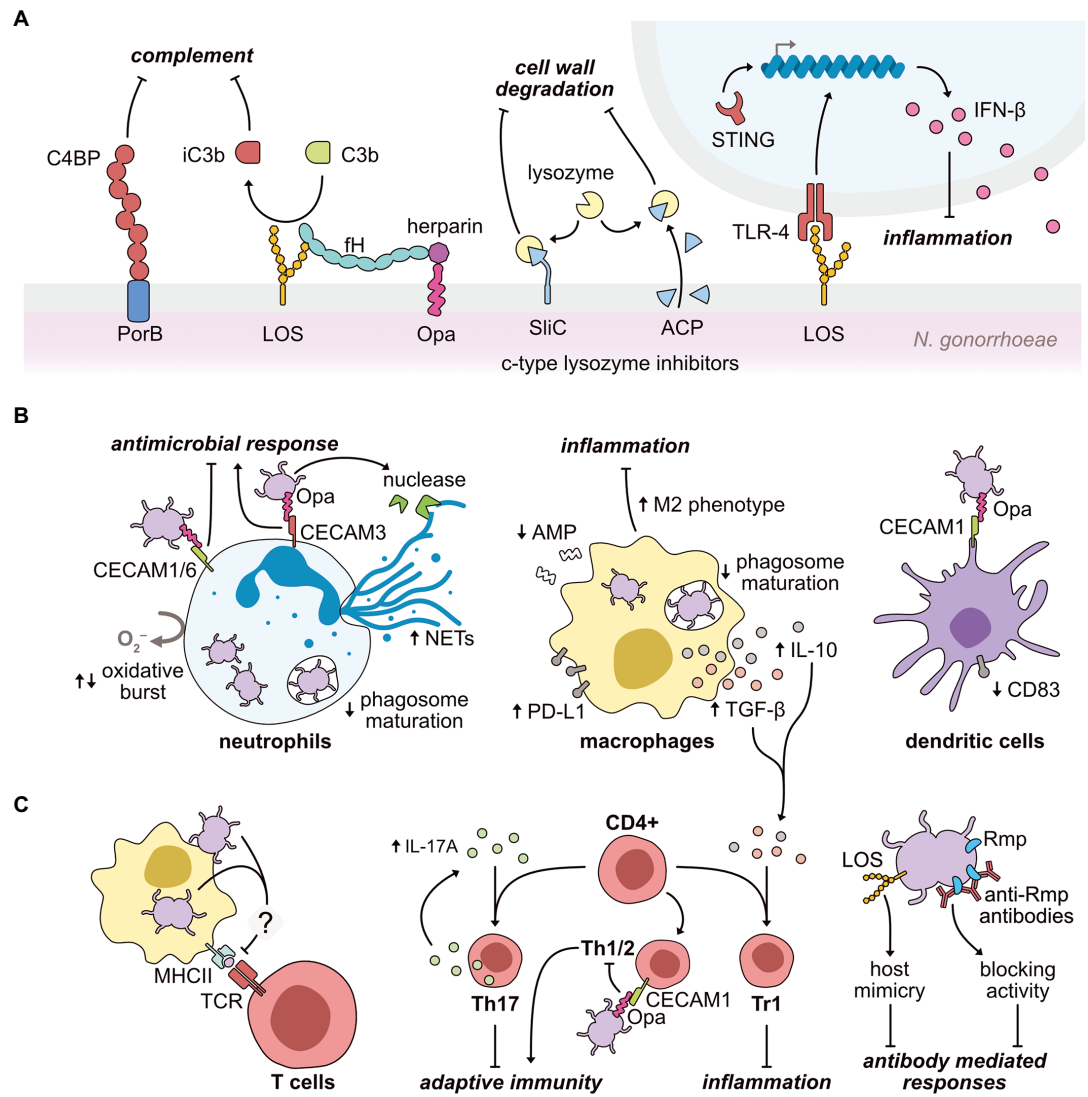
Modulation mechanisms exerted by *N. gonorrhoeae* on the host immune response during infection. **(A)** Gonococci use their primary outer membrane antigens to limit the efficacy of host lysozyme, complement, and pattern recognition systems. PorB and sialylated LOS interact with the soluble complement inhibitory proteins, C4BP and factor H (fH), respectively. LOS also binds to the soluble complement protein, C3b, inducing its inactivation (iC3b). Gonococcal Opa proteins further interact with host heparin to promote C3b inactivation. The gonococcus also expresses two surface-exposed lysozyme inhibitors (SilC and ACP), preventing the bactericidal activity of host lysozyme upon infection. In parallel, LOS interaction with the pattern recognition receptor, TLR-4, upregulates the expression of the anti-inflammatory cytokine, IFN-β. Gonococci-induced upregulation of IFN-β also occurs *via* activation of the cytosolic pattern recognition receptor, STING. **(B)** Gonococci repress antimicrobial functions of key innate effector cells to promote infection. The gonococcus engages with CEACAM receptors on neutrophils *via* its Opa proteins to repress (Opa-CEACAM1 and Opa-CEACAM6) or activate (Opa-CEACAM3) inflammatory responses, such as the oxidative burst. Neutrophil extracellular traps (NETs) are upregulated by gonococcal infection, although the secretion of a gonococcal nuclease may aid gonococci in resisting NET action. In both neutrophils and macrophages, gonococci are adept at delaying phagosome maturation to prolong intracellular survival. To specifically limit macrophage function, gonococci downregulates antimicrobial peptide (AMP) expression, while simultaneously upregulating immunosuppressive molecules to promote type 1 regulatory T (Tr1) cell proliferation. As part of this reprogramming, macrophages are polarized toward an anti-inflammatory M2 phenotype. Finally, Opa-CEACAM1 interactions on dendritic cells cause a downregulation of the CD83 maturation marker. **(C)** Gonococci act both directly and indirectly on the host adaptive immune system to minimize production of immunological memory. Gonococcal-challenged macrophages fail to increase major histocompatibility complex class II (MHCII) expression, which limits efficient antigen-presentation to cognate T cell receptors (TCRs). Instead, gonococcal infection upregulates the cytokine, IL-17A, directing CD4^+^ T cells toward a Th17 phenotype, less capable of inducing a protective response. Opa-CEACAM1 interactions on Th1/2 cells further reduces the efficacy of the adaptive immune response. Alongside the blunting of T cell-mediated immunity, B cell-mediated immunity is also minimized during gonococcal infection. Antibodies generated against the gonococcal Rmp protein block bactericidal effects of other circulating antibodies. Furthermore, upon sialylation *in situ*, the gonococcal LOS molecule mimics host structures, thus presenting as “self” to the immune system.

Throughout infection, the gonococcus has also evolved several intricate mechanisms to mitigate the impact of the host immune response (Figure 2). These evasion mechanisms limit the generation of a host protective immune response, leaving the host defenseless against reinfection and proving vaccine development difficult. Understanding in detail these intricate immune evasion mechanisms will greatly advance vaccine design, and also enable insight into the causes of asymptomatic infection. Throughout this section, we highlight the immune evasion mechanisms of the gonococcus in both cervical epithelial cells and immune cells. Lenz and Dillard have recently reviewed the interactions between the gonococcus and the immune system in the fallopian tubes (Lenz and Dillard, 2018), demonstrating both similarities and differences to the lower genital tract.

### 3.1. The innate immune response

#### 3.1.1. *Neisseria gonorrhoeae* expresses surface-exposed lysozyme inhibitors

The host innate immune system acts quickly upon the detection of an invading pathogen, and begins attacking components of the bacterial cell wall (Ragland and Criss, 2017). Peptidoglycan is targeted by lysozyme, which hydrolyzes the bonds holding the peptidoglycan subunits together. Various isoforms of lysozyme are distributed throughout the human genome (Irwin et al., 2011); consequently, bacterial pathogens have evolved to express several potent lysozyme inhibitors (Callewaert et al., 2012). Upon infection, gonococci are likely to encounter the human c-type lysozyme found on mucosal surfaces and secretions, and within the degradative granules of innate immune cells (Ragland et al., 2020). *N. gonorrhoeae* encodes two surface-exposed inhibitors of human c-type lysozyme (Figure 2A) – the adhesin complex protein (ACP; Hung et al., 2013; Humbert et al., 2017) and the surface-exposed lysozyme inhibitor of c-type lysozyme (SilC; Zielke et al., 2018). In the absence of SilC, gonococcal colonization of the mucosa of the murine female genital tract is significantly reduced (Zielke et al., 2018).

#### 3.1.2. Engagement of pattern recognition receptors can promote pathogenesis

Pattern recognition receptors (PRRs) are expressed by the host on both epithelial and innate immune cells, and are an additional first line defense against invading pathogens. These PRRs recognize evolutionarily conserved molecules produced by pathogens, commonly referred to as PAMPs, stimulating a cascade of signaling events to upregulate innate immune mechanisms. It is well established that gonococci are recognized by several PRRs upon infection, including toll-like receptors (TLR), nucleotide-binding oligomerization domain-like receptors (NOD), and stimulator of interferon genes (STING; Fichorova et al., 2001; Fisette et al., 2003; Kaparakis et al., 2010; Liu et al., 2010; Woodhams et al., 2013; Mavrogiorgos et al., 2014; Zhu et al., 2018). Activation of these receptors typically culminates in early NF-κB-dependent inflammatory responses, frequently seen in symptomatic infections. Although pro-inflammatory responses can enhance gonococcal pathogenesis (Fisette et al., 2003; Kaparakis et al., 2010; Mavrogiorgos et al., 2014; Lenz and Dillard, 2018; Quillin and Seifert, 2018; Lovett and Duncan, 2019), the direct activation of these PRRs and the subsequent effects on gonococcal pathogenesis have been seldom studied.

In one notable example, Andrade et al., demonstrated an upregulation of the anti-inflammatory cytokine, type 1 interferon-β (IFN-β), upon gonococcal invasion of both human and murine cells, which occurred *via* both STING- and TLR-4-dependent engagement (Andrade et al., 2016). Elevated levels of IFN-β suppressed both neutrophil and macrophage killing of gonococci. TLR-4 engagement general TLR-4 signaling is known to upregulate two distinct pro- and anti-inflammatory pathways. The anti-inflammatory pathway causes the upregulation of IFN-β (Figure 2A) *via* the IRF3 transcriptional regulator, which is activated upon TRAM/TRIF signaling. In contrast, several pro-inflammatory cytokines can be upregulated by two central transcription factors, NF-κB and/or activator protein-1, upon stimulation of MyD88 signaling by TLR-4 engagement (Molteni et al., 2016). Consequently, despite the activation of TLR-4 *via* the same ligand (LOS), it is unknown how gonococci-TLR-4 interactions appear to sway TLR-4 signaling toward an anti-inflammatory path. However, the subcellular localization of TLR-4 during activation may explain how these opposing responses are induced. MyD88 signaling becomes activated at the plasma membrane, in contrast, the anti-inflammatory pathway is initiated upon TLR-4 engagement within the endosome (Molteni et al., 2016).

#### 3.1.3. *Neisseria gonorrhoeae* exploits complement regulatory proteins for silent epithelial entry and immune masking

Working in concert with innate pattern recognition, are host proteins of the complement system: an enzymatic cascade that circulates in blood and permeates tissues to rapidly clear invading pathogens (Merle et al., 2015). The gonococcus has evolved to effectively counter various host complement proteins and significantly reduce complement activity (Figure 2A). Specifically, the gonococcal PorB protein binds both soluble C4b-binding protein (C4BP; Ram et al., 2001) and factor H (fH; Ram et al., 1998a), with sialylated LOS also capable of interaction with fH (Ram et al., 1998b). Both C4BP and fH are inhibitory components of the complement system, which prevent the recognition of host cells as foreign. Therefore, recruitment of these proteins enables the gonococcus to mask itself from complement-mediated attack. Further prevention of complement activity is enabled by the interaction of LOS with the soluble complement component, C3b, causing its inactivation (iC3b), again presenting the gonococci as “self” to the immune system (Edwards and Apicella, 2002). A secondary effect of the deposition of iC3b on the gonococcal surface is the promotion of host cell invasion *via* the host iC3b receptor, CR3. Engagement of this receptor by gonococci triggers increased CR3 expression within cervical epithelial cells, promoting further invasion of the host cell, without triggering a host inflammatory response given the immune silent-nature of this receptor (Edwards et al., 2001, 2002). Notably, CR3 is absent from male, but not female, urogenital epithelia, suggesting a potential link between the higher degree of asymptomatic infection in females compared to males. CR3 is also expressed by several immune cells, including neutrophils, macrophages, and T cells (Lamers et al., 2021), however, whether the gonococcus silently enters these immune cells *via* this receptor is unknown.

Furthermore, the gonococcus has evolved to take advantage of the host compound, heparin, to aid complement system evasion through interaction with its Opa proteins (Chen et al., 1995). Mucosal surfaces contain reasonably high levels of extracellular glycosaminoglycans, including heparin (Jackson et al., 1991), therefore it is likely that gonococci have access to these compounds upon infection (Chen et al., 1995). Extracellular heparin directly binds fH, increasing the affinity of the gonococcal surface for C3b (Figure 2A), further promoting the deposition of iC3b on the gonococcal surface (Meri and Pangburn, 1990). This ability to associate with heparin-like compounds is unique to *Neisseria* species. In addition, the Opa-heparin interaction acts as a bridge for further interaction with the human glycoprotein, vitronectin (Duensing and van Putten, 1998; Singh et al., 2010). Vitronectin is used by the host as a mechanism to prevent lysis by membrane attack complexes, and therefore provides another layer of immune avoidance when co-opted by the gonococcus.

#### 3.1.4. *Neisseria gonorrhoeae* establishes intracellular niches within neutrophils during infection

A well-established trait of gonococcal symptomatic infection is the rapid influx of high numbers of neutrophils to the site of infection, forming gonococci-neutrophil-rich exudates (Johnson and Criss, 2011; Palmer and Criss, 2018; Quillin and Seifert, 2018). Upon the detection of inflammatory signaling, neutrophils rapidly transmigrate from circulation into various layers of host tissue, and ultimately the mucosae (Rosales, 2020). Problematically for host immunity, *N. gonorrhoeae* can not only survive within neutrophils but also persist and replicate (Johnson and Criss, 2011; Zughaier et al., 2014; Gunderson and Seifert, 2015; Quillin and Seifert, 2018), clearly demonstrating an ability to effectively dampen intracellular bactericidal effects exerted by neutrophils (Figure 2B).

Generally, the first interaction between neutrophils and the gonococcus occurs between the CEACAM family of receptors (CEACAM1, CEACAM3, or CEACAM6) and the gonococcal Opa protein (69102). While CEACAM1 and CEACAM3, respectively, inhibit or activate neutrophil functions *via* cytoplasmic immunoreceptor tyrosine-based signaling motifs, CEACAM6 lacks a cytoplasmic domain (McCaw et al., 2004; Muenzner et al., 2010). Nonetheless, engagement of CEACAM6 induces inhibitory signaling similar to CEACAM1 (Schmitter et al., 2007). Consequently, gonococcal engulfment as a result of CEACAM1 and CEACAM6 engagement by Opa inhibits pro-inflammatory neutrophil effector functions (Sarantis and Gray-Owen, 2012). Conversely, CEACAM3 binding stimulates neutrophil effector functions (Schmitter et al., 2004), including degranulation and the oxidative burst response (Booth et al., 2003; Sarantis and Gray-Owen, 2012), as well as the stimulation of neutrophil influx (Sintsova et al., 2014). Thus, the nature of the Opa-CEACAM interaction during infection has been proposed as a key contributor to either the presence or absence of symptoms, particularly given that the presentation of symptoms in gonococcal infection is largely a result of tissue damage caused by neutrophil influx (Sintsova et al., 2014). Furthermore, Sintsova et al., demonstrated the requirement of CEACAM3 activation in the stimulation of these neutrophil antimicrobial actions (Sintsova et al., 2014). Despite the potential for inflammatory responses to aid pathogenesis, this potent inflammatory response caused by CEACAM3 engagement is thought not to be the case. Instead, the Opa-CEACAM3 interaction has been proposed as a consequence of human evolution to combat invading pathogens, which utilize non-stimulating CEACAM receptors to infect host cells (Bonsignore et al., 2020). Ultimately, it remains whether CEACAM3-dependent phagocytosis of gonococci into neutrophils allows the gonococcal cells to persist within these potent immune cells, or whether the gonococcus can only establish intracellular niches within neutrophils when engulfed *via* CEACAM1 or CEACAM6. Notably, several gonococcal isolates from disseminated infections did not possess binding-capacity for CEACAM3, unlike isolates that primarily cause localized infections (Roth et al., 2013). This suggests that CEACAM3 engagement is not utilized by the gonococcus to promote pathogenesis.

In addition to the Opa-CEACAM3 interaction promoting an oxidative burst response within neutrophils, the gonococcal PorB protein may induce a similar effect when integrated into the neutrophil cell membrane (Gray-Owen et al., 1997b; Lorenzen et al., 2000). The oxidative burst response culminates in the production of reactive oxygen species, which are produced either in the extracellular milieu *via* NADPH oxidase activity, or within the intracellular environment *via* myeloperoxidase. Notably, infection experiments with Opa-deficient gonococci failed to stimulate a neutrophil oxidative burst response (Rest et al., 1982; Virji and Heckels, 1986; Fischer and Rest, 1988; Criss and Seifert, 2008; Johnson and Criss, 2011), suggesting that PorB likely acts to enhance this Opa-dependent effect. Again, this finding may provide a link between the tendencies for asymptomatic infection to present more often in women compared to men, given that Opaless gonococci are primarily isolated from female infections, whereas, Opa-expressing gonococci are primarily isolated from male infections (Swanson et al., 1988; Jerse et al., 1994; Jerse, 1999; Johnson and Criss, 2011). Nevertheless, Gunderson et al., demonstrated a situational dependency of this gonococcal effect on neutrophil function (Gunderson and Seifert, 2015). At a high multiplicity of infection (MOI; >20), which is thought to better emulate infection, oxidative burst responses were suppressed, whereas the opposite response was seen at low MOIs (<20). Despite an anti-inflammatory state seen at high MOIs, the production of neutrophil extracellular traps (NETs) were still observed. However, gonococci appeared to be resistant to these NETs, which is thought to be a result of the gonococcal nuclease secretion, which has been shown to degrade the NET matrix (Juneau et al., 2015). In addition to the effects on the oxidative defense mechanism of neutrophils, following internalization, the gonococcus slows the maturation of the phagosome *via* its pilus and PorB proteins (Bjerknes et al., 1995; Lorenzen et al., 2000; Johnson and Criss, 2011, 2013; Palmer and Criss, 2018). Phagosomes are termed “mature” upon fusion with cytoplasmic granules containing potent antimicrobial compounds. Therefore, avoiding these components greatly enhances gonococcal intracellular survival within neutrophils.

#### 3.1.5. Macrophages are reprogrammed by invading gonococci

The ability of the gonococcus to survive within the hostile intracellular environment of neutrophils is certainly unique, but the infiltration of the longer-lived antigen-presenting cells of the immune system significantly aids gonococcal pathogenesis. The relationship between the gonococcus and antigen-presenting cells provides a critical link between gonococci infection and the adaptive immune system. The specific manipulation of both macrophages and dendritic cells (DCs) by the gonococcus serves to dampen the establishment of protective immune memory (Figure 2B). As a result, reinfection is common given the lack of population adaptive immunity (Zhu et al., 2011). Similar to neutrophils, upon invasion of macrophages, gonococci can also delay the maturation of the phagosome, subsequently promoting intracellular survival. Mosleh et al., demonstrated profound differences in various Rab GTPases (Rab4, Rab5, and Rab7) within human macrophage phagosomes treated with and without the gonococcal PorB protein (Mosleh et al., 1998). These Rab GTPases are known regulators of phagosomal maturation (Gorvel et al., 1991; Bucci et al., 1992), and consequently, phagosomes treated with PorB took longer to acquire late endocytic markers (Rab7 and cathepsin D) compared to untreated phagosomes, demonstrating the PorB-induced delay in phagosome maturation.

Upon establishment of macrophage infection, the gonococcus can actively suppress expression of numerous antimicrobial peptides (AMPs), including LL-37, HBD-1, and SLPI, where this suppression is correlated to epigenetic modification of the macrophage genome (Zughaier et al., 2020). The gonococcus constitutively expresses a histone deacetylase (HDAC)-like protein, at a four-fold higher rate under anaerobic conditions compared to aerobic, suggesting a primary role for this protein during host infection. Counterintuitively, this HDAC-like protein appears not to directly modify host DNA, and therefore acts *via* a yet to be identified mechanism. A second avenue by which the gonococcus directly modifies immune cell gene expression is by targeting the regulatory elements of iron availability in macrophages to combat the host iron-limiting defense (Zughaier et al., 2014). This innate immune defense carried out by macrophages can occur *via* both intra- and extra-cellular mechanisms of action. One key pathway is the expression of hepcidin, a hormone which limits iron uptake within the gut, while maintaining intracellular iron stores within macrophages. Macrophages can also generate their own siderophores to minimize access to free iron, and this process is highly dependent on the production of enterobactin by the enzyme BDH2. Gonococcal infection of both a murine (RAW264) and human (THP-1) macrophage cell line caused the overexpression of hepcidin and the downregulation of BDH2 expression, while also manipulating the expression of several other genes involved in the iron-limiting response of macrophages. Iron is essential for gonococcal survival (Zughaier et al., 2014), and therefore, the ability of the gonococcus to circumvent the iron-limiting defense of macrophages is critical to a successful infection.

Once the gonococcus has evaded the primary defense mechanisms exerted by macrophages, immunomodulatory mechanisms to alter macrophage-induced adaptive responses are initiated. The gonococcus suppresses critical T-cell responses through the induction of macrophage anti-inflammatory signaling (Ortiz et al., 2015; Château and Seifert, 2016; Escobar et al., 2018). However, conflicting reports have emerged that suggest gonococcal infection can induce both pro- and anti-inflammatory signaling in macrophages (Makepeace et al., 2001; Duncan et al., 2009; Feinen et al., 2010; Packiam et al., 2010; Zughaier et al., 2014; Ortiz et al., 2015). In part, these discrepancies are linked to differences in experimental cell models and require the development of more representative macrophage models of infection to resolve. Nevertheless, an interesting correlation noted in many bacterial infections is the link between chronic and persistent infection and an ability to reprogramme macrophages toward an M2 phenotype (Ortiz et al., 2015). The M2 phenotype of macrophages is strongly anti-inflammatory and stimulates tissue repair, whereas the opposing M1 phenotype favors an inflammatory response, characterized by the proliferation of T helper 1 (Th1) cells. Notably, a shift toward an M2 phenotype has been noted for gonococcal infection (Escobar et al., 2013; Ortiz et al., 2015). Elevated levels of immunosuppressive IL-10, TGF-β1, and PD-L1 were noted upon gonococcal challenge of both murine and human macrophages, despite increases in inflammatory IL-6. This cytokine profile is indicative of a T helper 17 (Th17) cell response, which is generally less capable of targeting intracellular pathogens. In addition, static levels of the cell surface co-stimulatory molecule, CD86, and the major histocompatibility complex class II, were noted within gonococci-infected macrophages, which are both involved in stimulating an adaptive immune response (Magee et al., 2012; Wieczorek et al., 2017). Taken together, these data suggest a reduced ability of gonococci-infected macrophages to stimulate a T cell response (Escobar et al., 2013; Ortiz et al., 2015). Likewise, a failure to raise expression levels of the antibody receptor, CD64, was also demonstrated upon gonococcal macrophage infection, and is proposed to weaken the antibody-dependent cellular cytotoxicity response against the gonococcus (Ortiz et al., 2015).

#### 3.1.6. Gonococcal antigens induce an anti-inflammatory phenotype in dendritic cells

Typically, dendritic cell responses to foreign antigens are pro-inflammatory in nature and promote both T cell development and maturation (Cabeza-Cabrerizo et al., 2021). However, in both human and murine DCs, gonococcal antigens induce an anti-inflammatory response (characterized by IL-10 and PD-L1 production), which results in reduced production of CD4^+^ T cells (Zhu et al., 2012; Yu et al., 2013). One mechanism by which *N. gonorrhoeae* is hypothesized to elicit this DC response is *via* interactions of the gonococcal Opa protein with CEACAM1 on DCs. In support of this, Yu et al., correlated the Opa-CEACAM1 interaction on DCs with a significant downregulation in the DC maturation marker, CD83 (Yu et al., 2013). Combined with the gonococcal-macrophage research, it is clearly evident that the gonococcus exerts very similar effects on the antigen-presenting cells of the innate immune system, with an overall reduced development of an adaptive T cell response.

#### 3.1.7. Pro- and anti-inflammatory cytokine responses are induced during gonococcal infection

Cytokines are master regulatory molecules, which coordinate immune defense upon infection. *N. gonorrhoeae* manipulates host cytokine signaling pathways, again creating a balance between pro- and anti-inflammatory states. The greatest perturbations are generally observed in the tumor necrosis factor (TNF), interferon (IFN), and interleukin (IL) cytokine signaling pathways, which generally culminate in the upregulation of an inflammatory state; however the induction of anti-inflammatory cytokines has also been noted (Fichorova et al., 2001; Kaparakis et al., 2010; Mavrogiorgos et al., 2014; Andrade et al., 2016; Château and Seifert, 2016; Płaczkiewicz et al., 2022). Although the majority of these studies have not provided direct links as to whether these inflammatory states promote gonococcal pathogenesis, a smaller number of studies demonstrated direct manipulation events on host cells by the gonococcus, ultimately influencing inflammation to promote infection.

An example unique to the gonococcus is the secretion of heptose-1,7-bisphosphate (HBP; Gaudet et al., 2015). During the synthesis of gonococcal LOS, HBP is secreted from the cell as a by-product and penetrates the host cytosol *via* endocytosis. Gonococcal HBP triggers inflammatory responses in many mammalian cell lines, including epithelial and immune cells. Importantly, this activation does not induce infected host cell death (Malott et al., 2013; Gaudet et al., 2015), suggesting that an HBP-induced inflammatory response promotes pathogenesis. An additional mechanism used by the gonococcus to directly influence inflammation is the upregulation of host long non-coding RNAs (lncRNAs). Płaczkiewicz et al., first demonstrated the upregulation of the lncRNA, MALATI, in epithelial cells in response to gonococcal challenge (Płaczkiewicz et al., 2022). This lncRNA reduces the expression of pro-inflammatory cytokines through the NF-κB pathway in macrophages (Wu et al., 2015; Zhao et al., 2016; Mao et al., 2017). In the same study, the lncRNA, RP11-510N19.5, was also upregulated in epithelial cells upon gonococcal challenge and can influence the expression of the chemokine, CCL20, which attracts lymphocytes *via* chemotaxis. Lastly, the lncRNA, ERICD, was also overexpressed, and is thought to be indirectly involved in the upregulation of the inflammatory TNFα molecule through RNA-mediated gene silencing.

Less well understood are the recent findings by Landig et al. that the gonococcus can interact with host Siglec receptors expressed by immune cells (Landig et al., 2019). Siglec receptors are one of several mechanisms utilized by the immune system to differentiate “self” from “foreign.” These receptors recognize sialic acids presented on host cells, and upon binding induce anti-inflammatory signaling pathways within the immune cell to prevent immune attack. Therefore, by interacting with these receptors, gonococci can present as “self” to the host immune system. Humans also possess Siglec receptors that culminate in pro-inflammatory responses upon engagement, and the gonococcus has demonstrated binding to both pro- and anti-inflammatory Siglecs (Landig et al., 2019). Although at a glance it appears contradicting, it is hypothesized that humans have evolved these inflammatory Siglecs as a mechanism to combat invading pathogens that were utilizing anti-inflammatory Siglecs to hide from the immune system. Whether this interaction of the gonococcus with the human pro-inflammatory Siglecs is a product of gonococcal evolution remains to be elucidated.

### 3.2. The adaptive immune response

Protective immunity against gonococcal infection is generally reported to be minimal or absent, even in symptomatic infections, and gonococcal re-infection is common in both women and men (Kasper et al., 1977; Buchanan et al., 1980; Hook et al., 1984; Plummer et al., 1989; Hedges et al., 1998b, 1999; Fox et al., 1999; Schmidt et al., 2001). Although the poor adaptive immune response is a consequence of innate immune evasion strategies, the gonococcus has evolved several additional strategies to specifically hinder the development of both B and T cell immunity (Figure 2C).

#### 3.2.1. *Neisseria gonorrhoeae* hinders antibody production and function

Several studies have reported antibody titers against the gonococcus both during and following infection, however, it has been proposed that the specificity and/or levels of these antibodies are insufficient for robust protection against re-infection (Kasper et al., 1977; Buchanan et al., 1980; Hook et al., 1984; Plummer et al., 1989; Hedges et al., 1998b, 1999; Fox et al., 1999; Schmidt et al., 2001). Antibodies generated against the gonococcal outer membrane protein, Rmp, during infection increase the likelihood of both ascension of the infection and re-infection (Plummer et al., 1993). A fraction of these anti-Rmp antibodies prevent the function of various bactericidal antibodies (Figure 2C; Joiner et al., 1985; Rice et al., 1986; Virji et al., 1987; Lovett and Duncan, 2019). Supporting this, sera from patients with disseminated gonococcal infection hinder the bactericidal effects of uninfected sera against the gonococcus *in vitro* (Allen Mccutchan et al., 1978; Rice and Kasper, 1982).

Molecular mimicry exhibited by gonococcal LOS upon sialylation *in situ* contributes to the reduced production and/or efficacy of anti-gonococcal antibodies (Figure 2C; Mandrell et al., 1988; Moran et al., 1996; Quillin and Seifert, 2018). Sialylated LOS moieties share significant structural similarities with glycosphingolipids predominantly found on human erythrocytes (Moran et al., 1996), allowing the pathogen to present as “self” to the immune system (Tan et al., 1986; Wetzler et al., 1992; Moran et al., 1996). The substrate(s) used by the gonococcus for enzymatic sialylation are not only found within the genital tract, but also the bloodstream and neutrophils (Apicella and Mandrell, 1989), allowing the gonococcus to evade humoral immune responses throughout different stages of the infection.

In addition to the role the gonococcal IgA1 protease has in promoting intracellular survival, this protease can also further reduce mucosal antibody levels by cleaving the hinge region of secretory IgA1 (Blake and Swanson, 1978; Johnson and Criss, 2011). However, despite extensive studies of the equivalent protein from meningococci (Brooks et al., 1992; Parsons et al., 2004; Tommassen and Arenas, 2017; Spoerry et al., 2021; Zhigis et al., 2021; Kilian et al., 2022), the role of the gonococcal IgA1 protease in antibody cleavage during infection has not been extensively studied. Although the IgA1 protease from vaginal washings in women with gonorrhoea demonstrated antibody cleavage activity *in vitro* (Blake et al., 1979), no activity has been noted *in vivo* to date (Hedges et al., 1998a).

#### 3.2.2. *Neisseria gonorrhoeae* induces a Th17 polarization in T cells

Another well-conserved immunomodulatory mechanism of the gonococcus is the promotion of a Th17 cell response at the expense of a Th1/Th2 cell response (Figure 2C) – a critical contributor to the lack of lasting adaptive immunity (Feinen et al., 2010; Liu et al., 2013, 2014). Th17 cells are a subset of CD4^+^ T cells, and primarily function to invoke innate immune defenses, which the gonococcus is clearly well armed to defend. In contrast, Th1/Th2 cells are primarily involved in stimulating an adaptive immune response (Feinen et al., 2010). Specifically, gonococcal infection induces the cytokine IL-17A (Gagliardi et al., 2011; Masson et al., 2014), a stimulator of Th17 cell differentiation, and not IL-12, which is required for Th1 cell differentiation (Ramsey et al., 1995; Naumann et al., 1997; Simpson et al., 1999). Intriguingly, the absence of TLR-4 *in vitro* minimizes the degree of Th17 polarization, suggesting that a TLR-4-LOS interaction may be responsible for the induction of IL-17A expression (Feinen et al., 2010). Moreover, IL-17A is expressed stably throughout the murine infection period, suggesting that gonococci may maintain this polarization throughout infection. IL-17A also increases the secretion of neutrophil-attracting chemokines, LIX and MIP-2α, from murine genital cells. Accordingly, removal of the IL-17A receptor prolonged infection and reduced neutrophil recruitment within the murine infection model.

Th17 polarization is further biased through the inhibitory Opa-CEACAM1 interaction on Th1/Th2 cells (Boulton and Gray-Owen, 2002; Pantelic et al., 2005; Feinen et al., 2010; Packiam et al., 2010; Liu et al., 2012). Interestingly, CEACAM1 engagement by gonococci on T cells, in contrast to all other CEACAM1-expressing cells, does not result in phagocytosis of the bacterial cells, but instead increases the level of CEACAM1 expression, allowing for stable Opa-CEACAM1 binding and prolonged inhibition (Lee et al., 2008). This difference may reflect the active recruitment of downstream effector molecules, SHP-1 and SHP-2, by gonococcal CEACAM1 interactions with T cells. In contrast, SHP-1 activity is suppressed upon gonococcal interactions with CEACAM1 in both macrophages and neutrophils (Hauck et al., 1999). Notably, the inhibitory Opa-CEACAM1 interaction also occurs with human B cells, although ultimately results in cell death (Pantelic et al., 2005) – an effect that has not been demonstrated for T cells. One consequence of this inhibitory signaling within T cells is a reduction in T cell proliferation, where CEACAM1 inhibition alone is able to exert these suppression effects, without any additional contributing factors (Boulton and Gray-Owen, 2002).

Alongside the Th17 response, an upregulation of the anti-inflammatory cytokines, IL-10 and TGF-β, by gonococcal infection stimulates the proliferation of immunosuppressive type 1 regulatory T (Tr1) cells (Imarai et al., 2008; Liu et al., 2012, 2013, 2014, 2017). Dendritic cells are the likely source of IL-10 overexpression upon interactions with the gonococcus, as discussed above (Zhu et al., 2012). Notably, when the action of TGF-β and IL-10 are inhibited, a protective immune response is generated in a gonococcal mouse model (Liu et al., 2012, 2014). Although, when IL-10 function alone is prevented, the Th17 response is not affected, demonstrating a TGF-β requirement for Th17 cell production. Separate infection experiments with an Opa^−^ gonococcal strain in murine cells *in vitro*, and TLR-4 deficient murine lymph node cells with wild-type gonococci, demonstrated reduced levels of IL-10 expression, suggesting a likely role for Opa and the LOS-TLR-4 interaction in this response; although these findings remain to be confirmed *in vivo*.

## 4. Manipulation of host cell death pathways

In addition to the host immune response, regulated cell death pathways are a crucial mechanism used by the host to alleviate infection. At the highest level, there are three main overarching categories of regulated cell death, defined by the morphological features associated with the death phenotype: apoptosis, autophagy, and necroptosis. These pathways are tightly controlled by complex interactions between pro-death and pro-survival signals, which coordinate to orchestrate cell fate, and, in some cases, elicit an immune response. The manipulation of these pathways by *N. gonorrhoeae* is an important virulence mechanism used to both establish and promote infection.

### 4.1. Apoptosis

**Figure 3:**
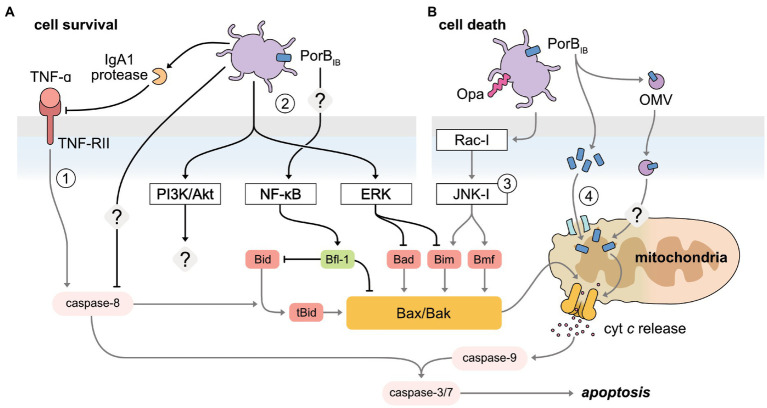
Manipulation of apoptosis pathways by *N. gonorrhoeae* to both inhibit and induce host cell death. **(A)** The receptor-mediated and mitochondrial-mediated apoptosis pathways are inhibited by gonococci to promote cell survival. (1) Secreted IgA1 protease has been shown to specifically cleave the TNFα conjugate receptor, TNF-RII, inhibiting receptor-mediated apoptosis. Caspase-8 is also inhibited, though the mechanism is undefined. (2) Host pro-survival pathways are activated upon gonococcal infection of host cells. NF-κB, activated through PorB_IB_ in an unidentified mechanism, increases the pro-survival protein Bfl-1, which inhibits the pro-apoptotic proteins, Bim and Bad. PI3K/Akt and ERK pathway activation is mediated by pili-expressing gonococci. The downstream impact of gonococcal-activated PI3K/Akt has not been investigated to date. Gonococcal-mediated activation of ERK downregulates the BH3-only proteins, Bim and Bad. **(B)** Gonococci promote apoptotic cell death by disrupting the integrity of the mitochondrial membrane. (3) Opa-expressing gonococci activate JNK-,1 through Rac-1, releasing BH3-only proteins, Bim and Bmf, sensitizing the mitochondria to permeabilization. (4) PorB is targeted to the inner mitochondrial membrane, disrupting the membrane potential to induce apoptosis. PorB can also be translocated to the mitochondria *via* delivery from outer membrane vesicles (OMV).

Apoptosis is the most well-characterized programmed cell death pathway, and is renowned for its seemingly immuno-silent nature given it elicits a minimal inflammatory response. Mammals possess two distinct, yet overlapping pathways, which lead to apoptotic cell death: the intrinsic (or mitochondrial-mediated) and extrinsic (or receptor-mediated) apoptotic pathways. These pathways are defined by a series of signaling events preceding the activation of the effector caspases (−3 and −7), which commit the cell to apoptosis by stochastically degrading cellular proteins. *N. gonorrhoeae* infection elicits both pro- and anti-apoptotic responses (Figure 3) in a variety of cell types, including epithelial cells of the urethra, cervix, and fallopian tubes, in addition to immune cells, such as macrophages and neutrophils (Muller, 1999, 2000, 2002; Beck and Meyer, 2000; Binnicker et al., 2003, 2004; Simons et al., 2005, 2006; Morales et al., 2006; Kepp et al., 2007, 2009; Reyes et al., 2007; Howie et al., 2008; Follows et al., 2009; Kozjak-Pavlovic et al., 2009; Chen and Seifert, 2011; Château and Seifert, 2016; Deo et al., 2018; Cho et al., 2020).

#### 4.1.1. *Neisseria gonorrhoeae* manipulates early events in the mitochondrial-mediated apoptotic pathway to both prevent and stimulate cell death

The defining event of the mitochondrial-mediated apoptotic pathway is the permeabilization of the mitochondrial membrane. This event is tightly regulated by members of the Bcl-2 family of proteins, comprising of three interacting protein groups: the anti-apoptotic proteins (Bcl-2), the pro-apoptotic proteins (BH3-only), and the pro-apoptotic effector proteins (Bax and Bak). Typically, cellular stress caused by bacterial infection results in the upregulation of the BH3-only proteins, which function to activate the pro-apoptotic effector proteins. This causes the membrane to permeabilize and subsequently releases cytochrome *c* into the cytoplasm, activating the caspase cascade. The gonococcus both subverts and accelerates this pathway *via* two main modes of manipulation — the secretion of PorB and the activation of host cell signaling pathways, ultimately affecting the balance of pro- and anti-apoptotic proteins (Muller, 1999, 2000, 2002; Binnicker et al., 2003, 2004; Simons et al., 2005, 2006; Kepp et al., 2007, 2009; Howie et al., 2008; Follows et al., 2009; Kozjak-Pavlovic et al., 2009; Chen and Seifert, 2011; Château and Seifert, 2016; Deo et al., 2018; Cho et al., 2020).

Prevention of mitochondrial-mediated apoptosis, specifically the early mitochondrial membrane permeabilization event, is observed in epithelial cells, neutrophils, and macrophages infected with high doses (MOI > 10) of *N. gonorrhoeae* (Binnicker et al., 2003; Simons et al., 2006; Chen and Seifert, 2011; Château and Seifert, 2016; Cho et al., 2020). While it is unknown how the MOI used in experimental settings emulates the different stages of infection, *N. gonorrhoeae*-mediated protection against apoptosis is thought to benefit pathogenesis by preserving replicative niches to facilitate both intra- and extra-cellular colonization and dissemination. In addition, prevention of apoptosis is yet another tactic employed by the gonococcus to overcome the antibacterial properties of innate immune cells.

*N. gonorrhoeae* infection of epithelial cells can activate several host pro-survival signaling pathways aimed at preserving the mitochondrial membrane potential. For example, the extracellular signal-regulated kinase (ERK) pathway and the PI3K/Akt pathway are activated upon infection of pili-expressing gonococci, in a manner that is enhanced by the physical force of the pili retraction motor (Lee et al., 2005; Howie et al., 2008). Although PI3K/Akt is a known negative regulator of mitochondrial-mediated apoptosis (Song et al., 2005), the downstream impact on apoptosis as a result of gonococcal-mediated PI3K/Akt activation has not been specifically investigated. Nevertheless, *N. gonorrhoeae*-mediated ERK activation results in the differential downregulation of the BH3-only proteins, Bim and Bad, *via* proteasome degradation and protein inactivation, respectively, in human T84 colonic epidermoid cells (Howie et al., 2008). Notably, inhibition of ERK in gonococcal-infected cells only partially restores the apoptosis phenotype of uninfected cells, suggesting that additional ERK-independent mechanisms are required for the full anti-apoptotic response *N. gonorrhoeae* elicits (Howie et al., 2008). In a separate instance, intracellular gonococci reduced ERK activation in transduced human urethral epithelial cells, which was correlated to a reduction in apoptosis. However, how this ERK-reduction leads to differences concerning the mitochondrial-mediated pathway was not investigated (Liu et al., 2015).

Alongside the ERK pathway, the gonococcus can also upregulate several pro-survival genes through the activation of NF-κB, including *bfl-1, cIAP-2, cox-2*, *mcl-1,* and *cFLIP* (Binnicker et al., 2003, 2004; Follows et al., 2009). However, only *bfl-1* and *cIAP-2* upregulation correspond to increases at the protein level in urethral and endocervical epithelial cells, respectively, suggesting a non-essential role for the remaining genes in *N. gonorrhoeae-*mediated apoptosis prevention (Binnicker et al., 2004; Follows et al., 2009). Bfl-1 is a member of the Bcl-2 pro-survival proteins, tasked with protecting mitochondrial membrane integrity by inhibiting the BH3-only protein, Bid, and effector protein, Bax (Zhang et al., 2000; Werner et al., 2002). In contrast, cIAP-2 belongs to the inhibitor of apoptosis proteins (IAPs), which were initially thought to only function as direct inhibitors of effector caspases. While this holds true for some IAP members, such as XIAP, more recent insight suggests other members, such as cIAP-2, offer additional functions, such as targeting components of the TNFα signaling pathway for ubiquitin degradation (Park et al., 2004). Intriguingly, the *N. gonorrhoeae*-stimulated increase of intracellular cIAP-2 was later determined to provide protection against a caspase-independent necroptosis-like cell death, as opposed to apoptosis, as caspase inhibition was observed to the same extent when cIAP-2 was experimentally deactivated (Nudel et al., 2015). This suggests that gonococcus-mediated activation of NF-κB may simultaneously modulate multiple cell death pathways, which is unsurprising given the wide array of signaling networks regulated by NF-κB. Furthermore, prolonged exposure of endocervical epithelial cells with live gonococci stimulated the production of exosomes for the extracellular exportation of cIAP-2, potentially as a mechanism to induce cell death at later time points in infection (Nudel et al., 2015).

In contrast to epithelial cell studies, the trend between gonococcal infection dose and the apoptotic outcome in neutrophils presents inconsistently. For example, increasing infection doses (such as MOIs between 1 and 100) have shown to both reduce and increase apoptosis inhibition, where the latter is in agreement with observations in epithelial cells (Simons et al., 2006; Chen and Seifert, 2011; Cho et al., 2020). Although the reasons for these discrepancies are unclear, previous studies highlight the importance of cell line choice in apoptotic outcome, suggesting that conflicting reports are likely valid (Binnicker et al., 2003; Château and Seifert, 2016). Nonetheless, the gonococcus is clearly able to subvert mitochondrial-mediated apoptosis in neutrophils, which constitutes an important antimicrobial mechanism used by these potent immune cells (Allen and Criss, 2019). Whether this mechanism of defense occurs in a similar manner to epithelial cells remains to be elucidated; although increased gene expression of the anti-apoptotic, *XIAP* and *cIAP-2*, is observed in gonococci-infected neutrophils, in parallel to depressed caspase activity (Simons et al., 2006), it has not been confirmed if this corresponds to increased protein levels, nor would this explain how the upstream event, mitochondrial membrane permeabilization, is prevented.

There are conflicting reports as to whether circumventing the mitochondrial-mediated pathway requires viable gonococci. Nonetheless, several studies have demonstrated partial apoptosis resistance after treatment of heat- or gentamicin-killed gonococci, leading researchers to hypothesize that gonococcal surface components are involved in apoptosis modulation (Binnicker et al., 2004; Simons et al., 2006; Château and Seifert, 2016). Binnicker et al., crucially demonstrated that purified PorB_IB_ at high doses (MOI equivalent of >10), but not other outer membrane components, such as pili, Opa proteins or LOS, was sufficient to upregulate pro-survival genes under the control of NF-κB activation in urethral epithelial cells. However, this was insufficient to confer the significant apoptosis-resistance observed for whole gonococci (Binnicker et al., 2004). This suggests that continued production of PorB_IB_ by viable gonococci could be important in maintaining resistance to apoptosis through an as yet undefined mechanism. Accordingly, treatment with PorB offers partial protection against apoptosis in endocervical epithelial cells but is dependent upon viable gonococci (Follows et al., 2009).

In striking contrast to infection at high doses, gonococcal infection of macrophages and epithelial cells at low doses (MOI of 1) induces apoptosis through similar manipulation strategies (Muller, 1999, 2000, 2002; Kozjak-Pavlovic et al., 2009; Deo et al., 2018). *N. gonorrhoeae*-induced apoptosis is postulated to promote infection by facilitating dissemination from initial sites of infection or as a means to evade death by executing macrophages. Integral to the mechanism of apoptosis-induction is the secretion of PorB; reliant on close contact with the host cell, PorB_IB_ translocates to the inner mitochondrial membrane, facilitated by existing mitochondrial import machinery (Kozjak-Pavlovic et al., 2009). Dependent upon ATP-binding, PorB subsequently lodges into the inner mitochondrial membrane, leading to a breakdown of mitochondrial membrane potential — a requirement for apoptosis induction (Kozjak-Pavlovic et al., 2009). Activation of host cell signaling pathways also plays a role in *N. gonorrhoeae*-induced apoptosis. Specifically, Opa-expressing *N. gonorrhoeae* mediate activation of the Rho family GTPase, Rac-1, stimulating a proliferation of activation events within the Jun-N-terminal Kinase-1 (JNK-1) signaling pathway, ultimately inducing the BH3-only proteins, Bim and Bmf, enabling the pro-apoptotic effector functions of Bak and Bax (Kepp et al., 2009). This, in combination with PorB sensitization, has been shown to stimulate gonococcal-induced apoptosis in epithelial cells (Kepp et al., 2007, 2009; Kozjak-Pavlovic et al., 2009). In macrophages, secreted outer membrane vesicles (OMVs) containing PorB have been found to facilitate PorB-targeting to the mitochondria, a mechanism sufficient to cause membrane permeabilization, cytochrome *c* release, and the downstream activation of effector caspases, all independent of the activation of host signaling pathways (Deo et al., 2018). Whether PorB dissociates from the OMV for transportation to the inner mitochondrial membrane *via* existing import machinery, as seen in epithelial cells, or if the OMV directly associates with the membrane to deliver PorB is not currently known. Although the latter explanation is currently favored given the observed “clustering” of PorB when associated with the mitochondria. Additionally, import through existing machinery requires unfolded protein, while PorB associated with OMVs adopts a similar native confirmation to that found in the outer membrane (Deo et al., 2018). It is interesting to note that OMV-facilitated delivery of PorB may be specific to macrophages based on the difference in the cell type responses to OMVs. In epithelial cells, OMVs are targeted to lysosomes for degradation (Bielaszewska et al., 2013), yet there is evidence OMVs may escape endosomes in macrophages and are detectable in the cytosol (Kozjak-Pavlovic et al., 2009; Vanaja et al., 2016), enabling the delivery of PorB. Intriguingly, the localization of PorB to the mitochondria appears to be a pathogenic-specific function of the *Neisserial* porin, as for commensal species, such as *Neisseria mucosa,* porin remains localized in the cytoplasm, further implicating the importance of PorB in pathogenesis (Muller, 2002).

#### 4.1.2. *Neisseria gonorrhoeae* manipulates early events in the receptor-mediated apoptotic pathway to both prevent and stimulate cell death

The receptor-mediated apoptosis pathway requires binding of death ligands to the corresponding death receptors, initiating caspase-8, and subsequently the effector caspases. Caspase-8 may also cross-communicate with the mitochondrial-mediated pathway through the cleavage of the BH3-only protein, Bid, where truncated Bid activates both Bax and Bak. In addition to its function as an immune inflammatory cytokine, the death signaling ligand, TNFα, initiates important signaling events within host cells that encounter *N. gonorrhoeae*, culminating in apoptosis to prevent colonization (Maisey et al., 2003; Morales et al., 2006; Yang et al., 2020). Epithelial cells of the fallopian tubes infected with gonococci at low doses (MOI of 1), induces TNFα-mediated apoptosis in adjacent cells (not associated with the bacteria), potentially as a means to disseminate from the initial sites of colonization and gain access to deeper tissue (Morales et al., 2006). However, at higher doses of gonococcal infection (MOI 10 and 100), the bacterium is able to circumvent TNFα-mediated apoptosis in cells of which it has direct association with, to colonize the fallopian tube epithelium, despite the presence of high TNFα concentrations. Based on these findings, Morales et al., postulate that an increase of an unidentified gonococcal product is responsible for protection against TNFα-induced apoptosis (Morales et al., 2006). Secretion of the IgA1 protease by the gonococcus upon infection of the monocytic cell line, U937, specifically cleaves the TNFα conjugate receptor, TRF-RII, but not TRF-RI, to inhibit apoptosis (Beck and Meyer, 2000). Given that TRF-RII is constitutively expressed in epithelial cells of the fallopian tube (Maisey et al., 2003; Reyes et al., 2007), it would be interesting to examine if the IgA1 protease contributes to apoptosis-inhibition in a similar manner to U937 cells. The hypothesis that the gonococcus acts early to inhibit the receptor-mediated apoptosis pathway is further supported by a study in U937 differentiated macrophages, where caspase-8 and subsequent Bid cleavage is inhibited in response to TNFα stimulated apoptosis (Château and Seifert, 2016), advantageously ceasing the communication between the two pathways for effective inhibition.

In addition to TNFα, the TNF-related apoptosis-inducing ligand (TRAIL) also functions as a death signaling ligand to initiate the receptor-mediated pathway. Inhibition of TRAIL-induced apoptosis has been demonstrated in neutrophils infected with gonococci. However, as apoptosis inhibition was determined by monitoring effector caspase-3 activation compared to uninfected cells, it is unclear if caspase inhibition was the mechanism of manipulation, or if the gonococcal targets lie upstream of this event, as has been postulated in macrophages (Chen and Seifert, 2011).

### 4.2. Autophagy

Autophagy is a complex, highly regulated, cytoprotective mechanism to control cellular homeostasis and adapt to metabolic stress to the benefit of the organism. To control infection upon intracellular pathogen invasion, autophagy mechanisms can be used by the host, by directly targeting the pathogen (here termed xenophagy), or by limiting their ability to survive through the elimination of critical replication factors. Regarding the former, autophagosomes target the invading pathogen and subsequently fuse with lysosomes for degradation, known as autophagy flux.

#### 4.2.1. *Neisseria gonorrhoeae* escapes xenophagy in epithelial cells and macrophages

Transcytosis is a prerequisite for disseminating gonococcal infection, during which intracellular gonococci are targeted by the autophagy pathway in epithelial cells through CD46-cyt1/GOPC signaling however a subpopulation of gonococci escape autophagy clearance (Lu et al., 2018). A recent mini-review published by Mendes et al., details current research on how *N. gonorrhoeae* escapes autophagy-mediated killing in epithelial cells (Mendes et al., 2020). In summary, the gonococcus possesses the ability to dampen CD46-cyt1/GOPC intracellular signals used by infected cells to initiate autophagy over time, and inhibit the maturation of autophagosomes and autophagy flux through the canonical pathway of activating an autophagy repressor complex, rapamycin complex 1 (Lu et al., 2018; Kim W. J. et al., 2019). A reduction of autophagic flux is also observed in murine macrophage and human macrophage-like cell lines, partially dependent upon phosphoethanolamine (PEA)-decorated lipid A (PEA-lipid-A)-expressing gonococci (Zughaier et al., 2015). While PEA-lipid-A is necessary for gonococcal survival when associated with phagocytes (Zughaier et al., 2015), how PEA-lipid-A enhances autophagy delay requires further investigation. Despite the conservation of autophagy machinery in neutrophils, and their involvement in antimicrobial activities (Shrestha et al., 2020), it has yet to be explored how the autophagic response in neutrophils is influenced during infection with *N. gonorrhoeae*.

### 4.3. Necroptosis-like cell death

Necrotic cell death pathways are distinctively characterized by rupture of the cell membrane. Originally thought to be a completely uncontrolled process, it is now known that necrosis has specific, programmed signaling events, referred to as necroptosis. Pyroptosis and pyronecrosis are two forms of necroptosis-like cell death, with similar inflammatory responses that are frequently directed against intracellular pathogens.

Pyroptosis is executed by the pore-forming protein, gasdermin D, following cleavage by caspase-1 or caspase-4 proteases. Caspase-1 is specifically activated by NOD-like receptor (NLR) inflammasome complexes, which forms in the cytosol upon the recognition of pathogenic stimuli (canonical pathway), or by caspase-4 which becomes activated following the direct binding of bacterial lipopolysaccharides (non-canonical pathway). Strong inflammatory responses are further mediated by caspase-1 maturation of IL-1β and IL-18 cytokines, which are released into the surroundings upon rupture. Pyronecrosis is predominantly activated through the NLR family pyrin domain containing 3 (NLRP3), forming an NLRP3-inflammasome, leading to cell death and the release of IL-1β and the pro-inflammatory factor, HMGB1, independent of caspase activation.

#### 4.3.1. *Neisseria gonorrhoeae* eliminates macrophages and propagates inflammatory responses by activating pyroptosis and pyronecrosis pathways

*N. gonorrhoeae* is a potent inducer of pyroptosis in monocyte-derived macrophages *via* both canonical and non-canonical pathways (Ritter and Genco, 2018). Nonetheless, the number of viable gonococci do not appear to be significantly impacted by pyroptosis, and it is hypothesized that the elimination of these phagocytic cells blunts the clearance of infection (Ritter and Genco, 2018). More recently, Li et al., determined induction of pyroptosis required viable gonococci rather than heat- or freeze/thaw-killed gonococci in a TLR-2-dependent manner (Li et al., 2019). At a mechanistic level, *N. gonorrhoeae* was found to prime NLRP3-inflammasome formation through the activation of NF-κB- and MAPK-dependent pathways, causing the increase in NLRP3 and proIL-1β transcription (Li et al., 2019). Various molecular signals that have been proposed to act upstream of NLRP3 activation and inflammasome formation are observed during gonococcal infection leading to caspase-1 activation (Li et al., 2019). In the absence of caspase activation, *N. gonorrhoeae* maintains the capacity to propagate an inflammatory response and cause cell death in monocytic THP-1 cells *via* pyronecrosis, dependent on cathepsin B activation. However, in either case the underlying gonococcal agonist has not yet been discovered (Duncan et al., 2009; Li et al., 2019).

## 5. Conclusion

The ability of the gonococcus to effectively colonize both the male and female urogenital tracts, with a diverse array of cellular structures requires a diversity of sophisticated infection strategies. Consequently, the gonococcus has evolved extensive capabilities to manipulate a wide range of host cell signaling pathways, dependent on both the site and progression of the infection. This review highlights that these manipulation events can favor opposing signaling cascades, such as either promoting or inhibiting the host inflammatory response or cell death pathways. Yet the balance between these opposing responses during infection remains largely unclear, in particular how these infection strategies may differentiate between both male and female infection, and symptomatic versus asymptomatic infections.

A common limitation highlighted throughout this review, and an unfortunate requirement to elucidate the aforementioned infection strategies, is the inability of current model systems to accurately reflect both the pathobiology and time-length of gonococcal infection. Animal models exclude human-specific components, immortalized cell lines do not truly mirror host expression profiles, and both organoids and primary cell culture lack extracellular influences (Edwards and Butler, 2011). Clearly, human volunteer studies are the optimal infection model to study, however, these studies are only able to be conducted in males, given the high risk of complications associated with female infection. Furthermore, male volunteer studies can only examine the early stages of infection, as ethically, treatment must be delivered upon the onset of symptoms.

The extensive ability of the gonococcus to elicit anti-inflammatory responses during infection is highlighted throughout this review. Interestingly, the acquisition of these responses is suggested as a defining event in the evolution of the commensals, *Neisseria lactamica* and *Neisseria polysaccharea*. These commensals are hypothesized to have evolved from the pathogenic *Neisseria* species, suggesting an evolutionary route from commensalism, to pathogenicity, and back to commensalism (Priniski and Seifert, 2018).

Overall, this review highlights the immense host manipulation capabilities the gonococcus possesses to promote pathogenesis, with additional mechanisms not covered in this review surrounding the direct manipulation of the host cell growth cycle (Jones et al., 2007; Vielfort et al., 2013; Weyler et al., 2014). Nevertheless, progress toward elucidating these infection strategies *in situ* will greatly support advancements in both prevention and treatment of this long-lasting human disease.

## Conflict of interest

The authors declare that the research was conducted in the absence of any commercial or financial relationships that could be construed as a potential conflict of interest.

## Publisher’s note

All claims expressed in this article are solely those of the authors and do not necessarily represent those of their affiliated organizations, or those of the publisher, the editors and the reviewers. Any product that may be evaluated in this article, or claim that may be made by its manufacturer, is not guaranteed or endorsed by the publisher.

## Author contributions

JH: concept, planning, writing, formatting, and funding. EW and SN: concept, planning, writing, and formatting. WK: planning, writing, formatting, proofing, and funding. KH: figures, formatting, and proofing. All authors contributed to the article and approved the submitted version.
